# Parental rejection and internalizing/externalizing problems among left-behind children: the moderating role of interpersonal harmony in class

**DOI:** 10.3389/fpsyg.2024.1385250

**Published:** 2024-11-26

**Authors:** Zixiao Liu, Zizheng Zhao, Huijing Chen

**Affiliations:** ^1^Department of Social Work, School of Sociology and Political Science, Shanghai University, Shanghai, China; ^2^Department of Neuroscience, Jockey Club College of Veterinary Medicine and Life Sciences, City university of Hong Kong, Kowloon, Hong Kong SAR, China

**Keywords:** internalizing problem, externalizing problem, parental rejection, class interpersonal harmony, left-behind children, moderation effect

## Abstract

**Background and objectives:**

Left-behind children are characterized by prolonged separation from one or both parents, which exposes them to a constant lack of good parenting, leading to increased risk of internalizing and (or) externalizing problems. This study explored the effects of parental rejection on internalizing and externalizing problems, and examined the moderating role of interpersonal harmony in class.

**Methods:**

The sample comprised 3,473 left-behind children (aged 6 ~ 15; 54.1% girls) in a rural area of southwest China. Self-reported measures including Parental Acceptance-Rejection Questionnaire, Class Interpersonal Harmony Questionnaire and Child Behavior Scale were administrated. Regression analysis was employed and Hayes PROCESS macro was utilized to test the moderation effect.

**Results:**

The analysis showed that parental rejection had a significant predictive effect on both internalizing problems (*β* = 0.33, *p* < 0.001) and externalizing problems (*β* = 0.40, *p* < 0.001) of the left-behind children. Teacher interpersonal climate (*β* = −0.05, *p* < 0.001) and peer interpersonal climate (*β* = −0.04, *p* < 0.01) significantly moderated the relationship between parental rejection and externalizing problems for children with both parents absence, but not for children with single parent absence.

**Conclusion:**

Benign teacher interpersonal climate and peer interpersonal climate may buffer the detrimental effect of parental rejection on left-behind children’s externalizing problems.

## 1 Introduction

China’s modernization and urbanization reforms have given rise to large waves of rural migrant workers, who move from remote rural to urban regions to seek for better employment opportunities and higher income. Considering the relatively high living expenses in urban areas and barriers to education and health care, these migrant workers typically leave their children behind (Wang and Hu, 2019). In Chinese academia, left-behind children are commonly defined as underage children (a) who are left in their rural hometowns with one or both parents moving to work in cities, and (b) who are raised and educated by only one parent or by grandparents, relatives, neighbors, or peers (Chai et al., 2019). According to statistical estimations, the total number of left-behind children in China with the absence of one or both parents exceeded 66 million, while over 45% children were left behind with both parents working in cities (Lyu et al., 2024). Since parents play an important role in children’s development of social skills and self-concept (Amato, 1993), the absence of parents during childhood may cause feelings of being neglected (Wen et al., 2019), impair children’s healthy growth (Fellmeth et al., 2018) and lead to higher rates of depression (Wang et al., 2015), anxiety (Dai and Chu, 2018; Zhao et al., 2014), and other psychiatric symptoms (Dai et al., 2017; Sun et al., 2017).

Internalizing and externalizing problems are two main categories in child psychopathology (Achenbach et al., 2016). Internalizing problems are inner-directed and generates disturbance within the individual, including anxiety, depression, social withdrawal or other emotional problems. On the contrary, externalizing problems are outer-directed and creates trouble and conflicts in the social environment, which typically includes conduct problems such as aggression, deviance, anger, hyperactivity and impulsivity (Achenbach et al., 2016). In general, left-behind children manifest more internalizing and externalizing problems compared to non-left-behind children (Fellmeth et al., 2018; Hu et al., 2018; Wang et al., 2019; Wang et al., 2015; Yu et al., 2022; Zhou et al., 2020). Moreover, internalizing and externalizing problems during childhood and adolescence tend to be persistent into adulthood (Cosgrove et al., 2010; Reef et al., 2011), and are highly connected with maladjustments later in life, such as more mental health problems (Dekker et al., 2007), lower life satisfaction and subjective happiness (Zhao et al., 2019), and functional impairments including lower educational attainment (Dekker et al., 2007), less academic achievement (Zhao et al., 2019) and work disability (Narusyte et al., 2017). Therefore, it is imperative to investigate potential risk and protective factors of internalizing and externalizing problems in left-behind children in order to shed light on evidence-informed interventions and policy formulations.

One major precipitant for emotional and behavioral disturbances among left-behind children is parental rejection, which is defined as intentional absence or withdrawal of parental warmth, love, or affection (Khaleque, 2015). It should be noted that parental absence does not always indicate parental rejection. For left-behind children, the absence of their parents is typically caused by the harsh reality of life that parents must migrate to big cities to earn higher income to support the family. Compared with children in intact families, left-behind children usually suffer more from long-term separation from their parents and the deprivation of parental participation in family education. In such cases, the parents are *unable* to provide sufficient parental love, which means it is not “withdrawal,” but rather restricted by reality. However, parental rejection is characterized by the *unwillingness* of parental involvement and acceptance. In other words, parental absence describes the painful external reality, while parental rejection emphasizes internal perception and the poor quality of parent–child relationship. Conceptually, unable and unwilling lack of parental love may have distinct implications for children’s development and adjustment. For example, Del Giudice (2009) proposed that the former one may lead to ambivalent attachment style, whereas the latter one may lead to avoidance attachment style.

Despite the fact that parental absence does not necessarily lead to perceived parental rejection, accumulating evidence shows that parental rejection is more prevalent among left-behind children (Adumitroaie and Dafinoiu, 2013; Yang et al., 2021). As a basic assumption of parental acceptance-rejection theory, a person’s psychological adjustment throughout life can be greatly influenced by experiences of parental acceptance and rejection, regardless of differences in race, gender, or culture (Rohner and Khaleque, 2010). In line with that, a longitudinal multicultural study showed that children’s perceived parental acceptance-rejection was associated with internalizing and externalizing problems across cultures (Rothenberg et al., 2022). Perceived parental rejection among left-behind children shapes an unstable “love without affinity” parent–child relationship (Xiao, 2022). Indeed, there is empirical evidence that parental rejection is associated with higher risk of both internalizing and externalizing problems among children in China (Ma et al., 2024; Zhang et al., 2022).

Although parental rejection may have detrimental impact on the mental health of left-behind children, a benign school environment could instead buffer its negative effect (Hung et al., 2023; Ling et al., 2017). Indeed, there is a growing interest in current literature to examine the impact of interpersonal factors at school on children’s internalizing problems (Murray et al., 2021; Wang and Liu, 2021; Yang et al., 2020; Zhu et al., 2024) and externalizing problems (Wang and Liu, 2021; Zhu et al., 2020). Findings from these studies point to the effect that positive relationships with teachers and peers at school can help prevent both internalizing and externalizing problems from developing or worsening in children (Murray et al., 2021; Yang et al., 2020; Zhu et al., 2020, 2024). Such buffering effect has also been observed among left-behind children. For instance, one previous study reported that perceived positive teacher-student relationship was negatively associated with both internalizing and externalizing problems among left-behind children (Liu et al., 2015). Similarly, one recent study found that teacher-student relationship and classmate relationship negatively predicted externalizing problems of left-behind students (Xie et al., 2020).

These findings can be well-understood within the frame of the stress-buffering model (Cohen and Wills, 1985) and the social ecological model (Bronfenbrenner and Ceci, 1994). According to the stress-buffering model, supportive social relationships can alleviate the detrimental impact of stressful events (such as parental rejection) on left-behind children (Cohen and Wills, 1985). On the other hand, the social ecological model describes a comprehensive picture to understand the social environment of left-behind children. It is postulated that a child is at the center surrounded by concentric circles comprised of five systems: microsystem, mesosystem, exosystem, macrosystem, and chronosystem (Bronfenbrenner and Ceci, 1994). At the microsystem and mesosystem levels, a child interacts with his/her family, peers and schools. In fact, previous official statistics showed that at the basic education stage (Grade 1 to Grade 9), there were 32.76 million boarding students in rural area in China, of which 60% were left-behind children (Huang, 2015), while a recent study reports similar statistical estimations (Wang et al., 2023). This indicates that a large proportion of left-behind children spend most of their time at school. Evidently, school is regarded as advantageous to address the social and emotional issues of left-behind children (Wang et al., 2017). Social support and relationship from school may compensate the parental rejection to a certain degree (Xie et al., 2020), which highlights the potential protective role of school to mitigate the inadequacy of parental involvement. Accordingly, China’s State Council proposed to improve the care and protection system for rural left-behind children, including families, schools, mass organizations, governments, and social forces (Ministry of Civil Affairs, 2023), in order to optimize left-behind children’s mental health.

Some researchers believe that the class is a fundamental source of children’s interpersonal relationships and various activities, and has an important influence on children’s mental health as a microsystem within the school system (Battistich et al., 2010; Eccles and Roeser, 2005). Compared with western school environments, Chinese schools place more emphasis on students actively participating in class collective activities and class management. Class is typically regarded as “the second home” for Chinese school students (Jiang, 2004). Therefore, the content of social interaction in the class environment is more intensive than the one in the west (Chen et al., 1995; Jiang, 2002). Chinese researchers therefore proposed the concept of class interpersonal harmony based on Chinese philosophy, which refers to the stability and positivity of class interpersonal relationships (Chen and Li, 2009). This concept has three sub-dimensions: teacher interpersonal climate, peer interpersonal climate and class structure, which fits well with the three aspects of class interpersonal interactions that have been explored in the west in terms of teacher-student relationships, peer relationships, and class structure (Battistich et al., 2010; Schaps et al., 2004). Conceptually, the first two dimensions, i.e., teacher interpersonal climate and peer interpersonal climate, correspond to the relational and affective dimension of the class environment, and intend to assess the extent of children’s perceptions that their teachers and classmates are supportive and caring. The third dimension, i.e., class structure, corresponds to the organizational dimension and measures the extent to which students have an active role in mutual decision making and norm setting (Battistich et al., 2010; Schaps et al., 2004). By definition, class structure reflects the extent to which a class is well ordered and organized, and clear in expectations of students’ roles.

Previous research has shown that a positive class climate can mitigate children’s emotional and behavioral problems (Buyse et al., 2008). For instance, the study by Liu et al. (2015) revealed that compared with non-left-behind children, the association between teacher-student relationship and internalizing problems was stronger among the left-behind children, indicating greater responsiveness to the protective effect of a desired teacher-student relationship. Ling et al. (2017) showed that higher friendship quality and peer acceptance among left-behind children were highly correlated with lower sense of loneliness, which is one of the important predictors of both internalizing and externalizing problems (Hu et al., 2018). Similarly, Xie et al. (2020) demonstrated that good teacher-student relationships and peer relationships were conducive to reducing loneliness and to preventing problematic behaviors of left-behind students. On the contrary, a lack of positive emotional connections between teachers and students may be associated with a variety of externalizing problems such as stealing, aggression, smoking, and alcohol abuse in early adolescents (Rudasill et al., 2010; Silver et al., 2005).

With regard to the organizational dimension, direct evidence is rather sparse to illustrate the influence of class structure on internalizing and (or) externalizing problems among left-behind children. For example, in a study by Li et al. (2013), which used the same measurement developed by Chen and Li (2009), the authors reported negative correlation between the total score of class interpersonal harmony and externalizing problems, without presenting results from each of the three subscales. On the other hand, there is indirect evidence showing that class structure is negatively associated with bullying behaviors (Li M. et al., 2015) and positively associated with prosocial behaviors (Chen and Li, 2009). Moreover, studies in Western countries indicate that a well-established class structure may be related to children’s healthy development and well-being (Modin and Östberg, 2009; Rathmann et al., 2018; Samdal et al., 1998). In sum, the link between the dimension of class structure and internalizing/externalizing problems in left-behind children awaits further examination.

Taken together, although there is substantial evidence indicating the protective effect of class interpersonal harmony, it remains unexamined how parental rejection and class interpersonal harmony interacts and how they are related to internalizing and externalizing problems of left-behind children. Hypothetically, combining the perspective of the social ecological model and the stress-buffering model, for children who have more resources in their meso-system, i.e., better teacher-student relationship and peer relationship and more positive class structure, parental rejection in their micro-system may have a less harmful effect. In other words, left-behind children who perceive more class interpersonal harmony may be at lower risk of developing internalizing and externalizing problems, despite of their perceived parental rejection. Moreover, the type of parental absence (i.e., single parent absence or both parents absence) might also have an impact.

**Figure 1:**
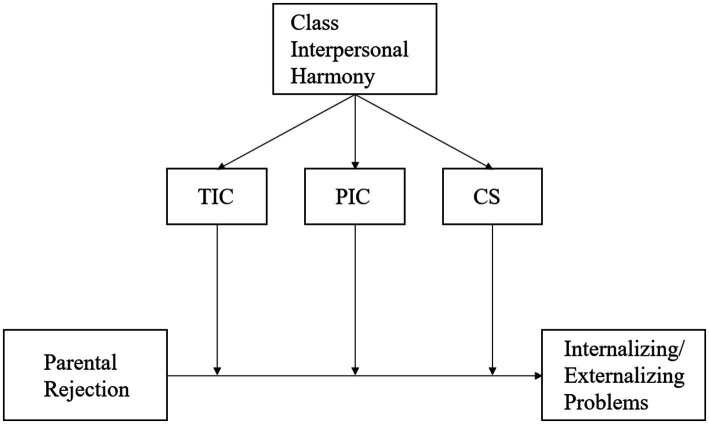
Conceptual model. TIC, teacher interpersonal climate; PIC, peer interpersonal climate; CS, class structure.

Therefore, the present study aims at investigating the potential moderating role of class interpersonal harmony in the association between perceived parental rejection and internalizing/externalizing problems among left-behind children in China. The effect of type of parental absence will also be examined. The following hypotheses are formulated: (1) parental rejection is positively associated with children’s internalizing and externalizing problems; and (2) classroom interpersonal harmony can moderate the effects of parental rejection on left-behind children’s internalizing and externalizing problems. The conceptual model of the present study was depicted in Figure 1.

## 2 Materials and methods

The present study received ethical approval from the Ethics Committee of the School of Humanities of Tongji University.

### 2.1 Participants and procedures

This cross-sectional study was conducted in a rural area in Yunnan Province, which is located in southwest China as one of the major migrant-sending areas in China. Official statistics show that there are over 1 million rural left-behind children in Yunnan (National Bureau of Statistics of China, UNICEF China, & UNFPA China, 2023). Three thousand five hundred and sixteen questionnaires were randomly distributed to left-behind children in 160 primary and secondary schools (Grade 1 to Grade 9). The questionnaires were all administered on site by psychometrically trained village staff. The subjects voluntarily participated in this survey by filling out the questionnaire after providing written informed consent. As the survey involved minimal risk, informed consent by parents or legal guardians was waived. After excluding questionnaires that were obviously disorganized (all choosing one answer, or answering in sequence, or answering in S-shape) or incomplete (answering less than 80% items), a total of 3,473 (98.8%) valid questionnaires were included in the analysis.

Among the 3,473 valid questionnaires in this study, 1,595 (45.9%) were males and 1878 (54.1%) were females. The median age was 12.0 years (interquartile range = 1.5, range 6.0 ~ 15.0). Among them, 183 (5.3%) were left-behind children whose mothers went out alone, 457 (13.2%) were left-behind children whose fathers went out alone, and 2,833 (81.6%) were left-behind children whose both parents went out. Table 1 presented the demographic characteristics of the sample.

**Table 1:** Demographic characteristics (*N* = 3,473).

Variables	*n* (%)
Grade	
Grade 1 to Grade 6	2,275 (65.5)
Grade 7 to Grade 9	1,198 (34.5)
Sex	
Male	1,595 (45.9)
Female	1,878 (54.1)
Parental absence	
Only mother	183 (5.3)
Only father	457 (13.2)
Both parents	2,833 (81.6)
Only child	
Yes	934 (26.9)
No	2,539 (73.1)

### 2.2 Instrument

#### 2.2.1 Parental acceptance-rejection questionnaire

The parental rejection dimension of the short version of the Parental Acceptance-Rejection Questionnaire for Children and Adolescents developed by Rohner and Khaleque (2005) was used to examine the perceived parental rejection behavior of left-behind children. The parental rejection dimension of the questionnaire consists of 16 questions, which are scored on a 4-point Likert scale ranging from 1 “almost never true” to 4 “almost always true.” The mean score of the 16 questions was used, with higher scores indicating higher levels of perceived parental rejection. Studies have shown that the Chinese version of the questionnaire has good reliability and validity and is applicable to the Chinese cultural background (Chen et al., 1995). Since a large proportion of left-behind children only have one primary caregiver (Liu et al., 2017), the father and mother rejection dimensions were combined into one dimension of parental rejection. The internal consistency coefficient of the parental rejection dimension in this study was 0.81.

#### 2.2.2 Class interpersonal harmony questionnaire

The Student Perceived Class Interpersonal Harmony Questionnaire, developed by Chen and Li (2009), was used to examine the perceived interpersonal harmony in the classroom of the left-behind children. The questionnaire consists of 20 questions and contains three dimensions: teacher interpersonal climate (consisting of 7 questions), peer interpersonal climate (consisting of 6 questions), and class structure (consisting of 7 questions). Example questions of the three dimensions are as follows: “We get along well with our teachers” (teacher interpersonal climate), “Classmates will support and encourage each other” (peer interpersonal climate), and “Our class is like a big family and is warm “(class structure). The questionnaire was scored on a 5-point Likert scale, ranging from 1 “never like this” to 5 “always like this.” Higher scores indicate greater class interpersonal harmony perceived by the children in the class. The questionnaire refers to the class environment theory by Schaps et al. (2004) and the Chinese class environment measurements (Guo, 2004; Jiang, 2002). It has been shown to have good reliability and validity for the Chinese cultural context. The average scores of each of the three subscales were used accordingly. In this study, the internal consistency coefficients of teacher interpersonal climate, peer interpersonal climate, and class structure were 0.83, 0.82, and 0.77, respectively.

#### 2.2.3 Children’s behavior scale

The short version of the Child Behavior Scale revised by Li et al. (2009) was used, which was derived from the Child Behavior Checklist developed by Achenbach (1991). The questionnaire consists of 40 questions, including two dimensions: internalizing problems (consisting of 16 questions) and externalizing problems (consisting of 24 questions). The former refers to emotional problems such as anxiety, depression, and withdrawnness, and the latter refers to behavioral problems such as aggression, defiance, disciplinary transgressions, and hyperactivity. Examples of the two dimensions are: “I feel lonely” (internalizing problems) and “I like to argue with others” (externalizing problems). In terms of scoring, Li et al. (2009) adopted a 4-point scale of 1–4, while Achenbach (1991) adopted a 3-point scale of 0–2. The present study used a more widely used 3-point scale of 0 (not true) to 2 (very true). This questionnaire has been shown to have good reliability and validity and is applicable to the Chinese cultural context (Shi et al., 2022). In this study, the internal consistency coefficients of internalizing problems and externalizing problems were 0.84 and 0.88, respectively.

### 2.3 Data analysis

Data were analyzed using the SPSS v.19.0 statistical software package, with the significance level set at *α* < 0.05 (two tails). Descriptive statistics and independent-sample *t*-tests for study variables grouped by gender were calculated. Pearson’s product correlation analysis was performed to examine the association between the main study variables. Regression analysis was performed for parental rejection and class interpersonal harmony on internalizing/externalizing problems. The three dimensions of class interpersonal harmony, i.e., teacher interpersonal climate, peer interpersonal climate and class structure were put into the regression model separately. Moderation analysis was performed using multivariate regressions with Model 1 of the SPSS PROCESS 3.4.1 macro (Hayes, 2017), using parental rejection as the independent variable, internalizing/externalizing problems as the dependent variable, and teacher interpersonal climate, peer interpersonal climate and class structure as moderating variables, respectively. Bonferroni corrections were applied for the significance level. Considering the parallel tests, a conservative alpha level was adopted with α = 0.01, since a *p*-value of below 0.0167 (= 0.05/3) is considered statistically significant.

Potential confounding variables were controlled, including sex, type of parental absence and status of being only child. Moreover, in order to examine the possible effect of type of parental absence, we further tested the moderating effect in children with both parents absence and in children with only father/mother absence, separately. The independent and moderating variables were centered to reduce multicollinearity in the moderation analysis. The simple slopes of the variables involved in the moderation models were plotted based on the mean ± 1 standard deviation of the moderation variables and the independent variables using the pick-a-point approach.

## 3 Results

### 3.1 Descriptive statistics and gender differences

We calculated the mean and standard deviation for parental rejection, teacher interpersonal climate, peer interpersonal climate, class structure, internalizing problems and externalizing problems, and compared differences by gender (Table 2). The results showed that male left-behind children perceived significantly higher parental rejection (*F* = 19.82, *p* < 0.001), lower peer interpersonal climate (*F* = 10.64, *p* < 0.01) and lower class structure (*F* = 7.31, *p* < 0.01), compared to their female counterparts. In addition, the externalizing problems (*F* = 35.18, *p* < 0.001) of male left-behind children were significantly higher than that of female left behind children. Teacher interpersonal climate and internalizing problems did not show significant gender differences (Table 1).

**Table 2:** Descriptive statistics and gender differences (*N* = 3,473).

Variables	Total (*M* ± *SD*)	Male (*M* ± *SD*)	Female (*M* ± *SD*)	*t*
Parental rejection	1.55 ± 0.45	1.58 ± 0.46	1.51 ± 0.44	4.43***
Teacher interpersonal climate	3.91 ± 0.79	3.88 ± 0.81	3.93 ± 0.78	−1.81
Peer interpersonal climate	3.69 ± 0.79	3.64 ± 0.81	3.73 ± 0.78	−3.26**
Class structure	3.56 ± 0.74	3.52 ± 0.76	3.59 ± 0.73	−2.70**
Internalizing problems	0.50 ± 0.33	0.49 ± 0.32	0.51 ± 0.33	−1.74
Externalizing problems	0.37 ± 0.27	0.40 ± 0.28	0.34 ± 0.25	5.88***

**p < 0.01, ***p < 0.001.

### 3.2 Correlation between study variables

Table 3 showed the correlation between the main study variables. Parental rejection was significantly and negatively correlated with teacher interpersonal climate (*r* = −0.29, *p* < 0.01), peer interpersonal climate (*r* = −0.25, *p* < 0.01) and class structure (*r* = −0.22, *p* < 0.01). Moreover, higher parental rejection was significantly correlated with more internalizing problems (*r* = 0.32, *p* < 0.01) and externalizing problems (*r* = 0.39, *p* < 0.01). Both internalizing problems and externalizing problems were significantly and negatively correlated with all three subscales of class interpersonal harmony (*r* = −0.26 ~ −0.33, all *p* < 0.01). Finally, internalizing problems and externalizing problems were highly associated (*r* = 0.66, *p* < 0.01).

**Table 3:** Correlation between study variables.

Variable	1	2	3	4	5	6
1. Parental rejection	1					
2. Teacher interpersonal climate	−0.29**	1				
3. Peer interpersonal climate	−0.25**	0.63**	1			
4. Class structure	−0.22**	0.68**	0.75**	1		
5. Internalizing problems	0.32**	−0.26**	−0.30**	−0.29**	1	
6. Externalizing problems	0.39**	−0.33**	−0.28**	−0.30**	0.66**	1

**p < 0.01.

### 3.3 Regression analysis

Table 4 showed that in the regression analysis, parental rejection had a significant positive effect on both internalizing and externalizing problems (both *β* =0.23, *p* < 0.001). The teacher interpersonal climate, peer interpersonal climate and class structure all had a significant negative effect on internalizing and externalizing problems (*β* = −0.13 ~ −0.10, all *p* < 0.001).

**Table 4:** Regression analysis of parental rejection and class interpersonal harmony on internalizing/externalizing problems.

The regression equation		Overall fitting index		Significance of regression coefficient	
Outcome	Predictor	*R* ^2^	*F*	*β*	*t*
Externalizing problems					
	PR	0.16	632.58	0.23	25.15***
	TIC	0.11	424.90	−0.11	−20.61***
	PIC	0.08	301.37	−0.10	−17.36***
	CS	0.09	335.77	−0.11	−18.32***
Internalizing problems					
	PR	0.11	390.05	0.23	19.75***
	TIC	0.07	257.99	−0.11	−16.06***
	PIC	0.09	349.20	−0.12	−18.69***
	CS	0.08	314.92	−0.13	−17.75***

PR, parental rejection; TIC, teacher interpersonal climate; PIC, peer interpersonal climate. ***p < 0.001.

### 3.4 Moderation analysis

We tested the moderating effects with model 1 of SPSS PROCESS 3.4.1 macro (Hayes, 2017), using centered score of parental rejection as the independent variable, internalizing/externalizing problems as the dependent variable, and centered score of teacher interpersonal climate, peer interpersonal climate and class structure as moderating variables, respectively. Moreover, considering the potential influences, we also controlled for variables including sex, type of parental absence and status of being only child. The results showed that teacher interpersonal climate and peer interpersonal climate moderated the association between parental rejection and externalizing problems.

Table 5 showed that in the moderation model, greater parental rejection was significantly associated with more externalizing problems. The interactions between parental rejection and teacher interpersonal climate (*β* = −0.05, *p* < 0.001), and between parental rejection and peer interpersonal climate (*β* = −0.04, *p* < 0.01) both had a significant negative effect on externalizing problems.

**Table 5:** Moderation analysis with the whole sample (*N* = 3,473).

The regression equation		Overall fitting index		Significance of unstandardized coefficient	
Outcome	Predictor	*R* ^2^	*F*	*β*	*t*
Model 1					
Externalizing problems		0.22	306.86***		
	PR			0.19	19.66***
	TIC			−0.08	−14.45***
	PR * TIC			−0.05	−3.95***
Model 2					
Externalizing problems		0.20	278.55***		
	PR			0.21	21.48***
	PIC			−0.07	−12.38***
	PR * PIC			−0.04	−3.09**

PR, parental rejection; TIC, teacher interpersonal climate; PIC, peer interpersonal climate. ***p < 0.001, **p < 0.01.

Given the relevance of type of parental absence, we also analyzed the moderation model separately for each of the three types of parental absence: only mother absence, only father absence, and both parents absence. The results showed that for left-behind children with both parents absence, the outcome of moderation model was consistent with the one using the whole sample. However, for left-behind children with only mother absence or only father absence, none of the three dimensions (i.e., teacher interpersonal climate, peer interpersonal climate and class structure) of class interpersonal harmony played a significant moderating role between parental rejection and internalizing/externalizing problems.

Table 6 showed that in the moderation model for left-behind children with both parents absence, greater parental rejection was significantly associated with more externalizing problems. The interactions between parental rejection and teacher interpersonal climate (*β* = −0.05, *p* < 0.001), and between parental rejection and peer interpersonal climate (*β* = −0.04, *p* < 0.01) both had a significant negative effect on externalizing problems.

**Table 6:** Moderation analysis for left-behind children with both parents absence (*n* = 2,833).

The regression equation		Overall fitting index		Significance of unstandardized coefficient	
Outcome variable	Predictor variate	*R* ^2^	*F*	*β*	*t*
Model 1					
Externalizing problems		0.22	251.35***		
	PR			0.19	17.11***
	TIC			−0.09	−13.99***
	PR * TIC			−0.05	−4.01***
Model 2					
Externalizing problems		0.20	225.12***		
	PR			0.21	18.53***
	PIC			−0.08	−12.11***
	PR * PIC			−0.04	−2.74**

PR, parental rejection; TIC, teacher interpersonal climate; PIC, peer interpersonal climate. ***p < 0.001, **p < 0.01.

**Figure 2:**
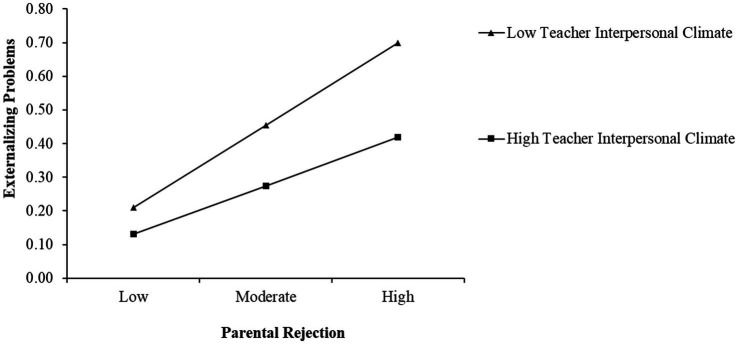
Teacher interpersonal climate as a moderator of the association between perceived parental rejection and externalizing problems.

**Figure 3:**
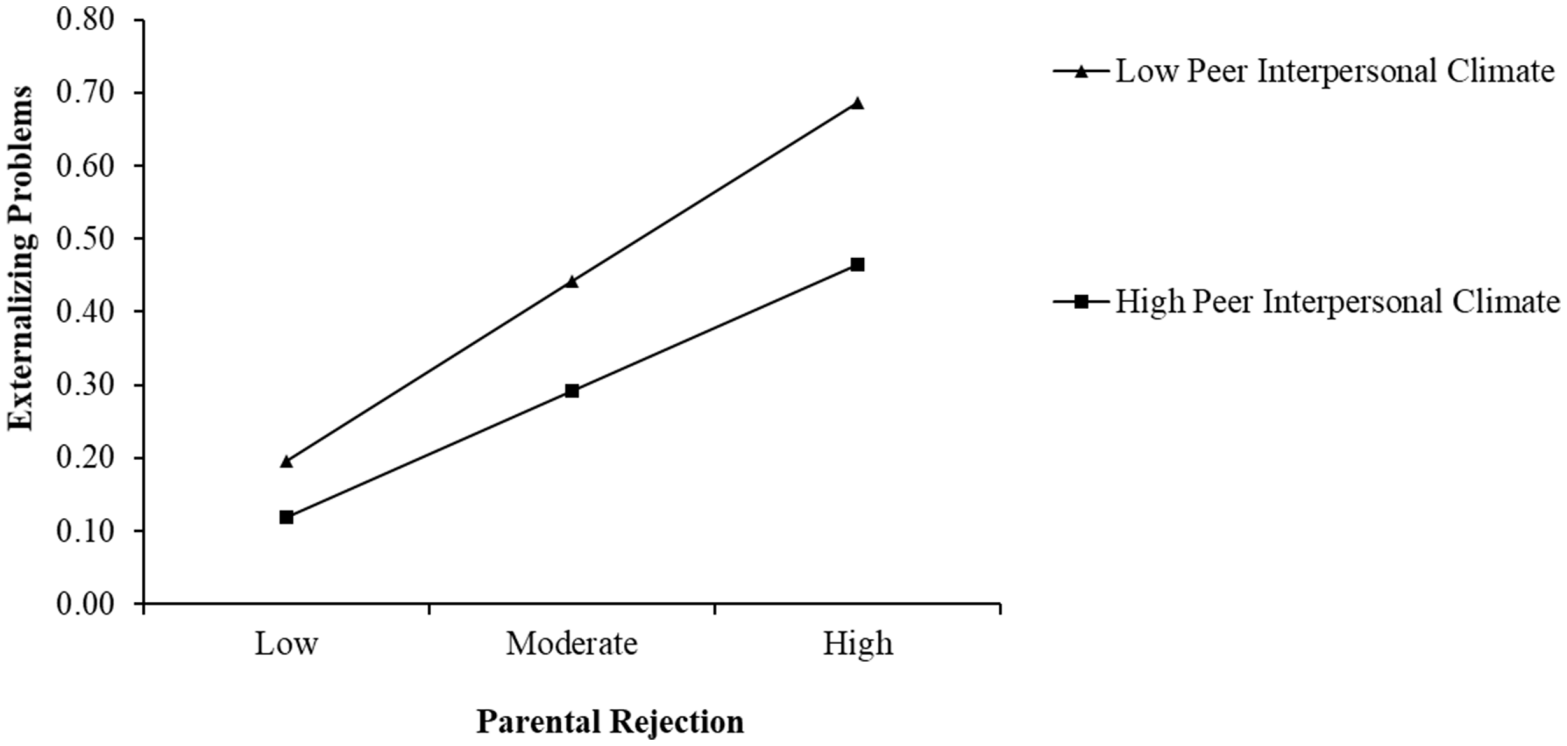
Peer interpersonal climate as a moderator of the association between perceived parental rejection and externalizing problems.

Since the moderating effects were significant only for left-behind children with both parents absence, we used this subgroup to plot simple slopes. The simple slope tests showed that the influence of parental rejection on externalizing problems was weakened for left-behind children with high teacher interpersonal climate, and high peer interpersonal climate (1 SD above the mean) compared to those with low teacher interpersonal climate, and low peer interpersonal climate (1 SD below the mean), as depicted in Figures 2, 3. Table 7 showed the results of the simple slope test.

**Table 7:** Testing the moderation effect.

Level of moderator	*B*	SE	*t*	LLCI	ULCI
Low TIC	0.23	0.01	16.69 ***	0.20	0.26
High TIC	0.15	0.02	8.94 ***	0.12	0.18
Low PIC	0.23	0.01	16.41***	0.21	0.26
High PIC	0.18	0.02	10.92***	0.15	0.21

TIC, teacher interpersonal climate; PIC, peer interpersonal climate. ***p < 0.001.

## 4 Discussion

The present study used a cross-sectional design to investigate the effects of parental rejection on internalizing/externalizing problems among left-behind children in China and the potential moderating role of class interpersonal harmony. Self-reported measures including Parental Acceptance-Rejection Questionnaire, Class Interpersonal Harmony Questionnaire and Child Behavior Scale were assessed with a sample of 3,473 left-behind children in a rural area of southwest China. The results of the current study are partially in line with our hypotheses. In other words, we found that (1) parental rejection was significantly and positively associated with both internalizing and externalizing problems; and (2) both teacher interpersonal climate and peer interpersonal climate moderated the effects of parental rejection on left-behind children’s externalizing problems. The relevance and implications of our findings are discussed as below.

First of all, the results supported our hypothesis I. Our finding indicates that left-behind children who perceived more parental rejection were emotionally and behaviorally more disturbed, which is consistent with previous research (Akse et al., 2004) as well as the parental acceptance-rejection theory (Rohner and Khaleque, 2010). Theoretically, parental rejection may have a lifelong substantial impact on children’s psychological and behavioral adaptation (Rothenberg et al., 2022). Indeed, constant emotional deprivation and insufficient relational support may impair left-behind children’s healthy development of emotion regulation and social skills, causing underdeveloped self-control (Zhang et al., 2022), deficient executive function (Ma et al., 2024) and increased deviant peer affiliations (Yang et al., 2021), thus leading to higher risks of developing both internalizing and externalizing problems.

Moreover, in the correlation analysis, we found that parental rejection was negatively correlated with all three sub-dimensions of interpersonal harmony in class: teacher interpersonal climate, peer interpersonal climate and class structure. This suggests that the left-behind children who perceived more parental rejection also experienced lower level of interpersonal harmony in the class. In addition, the three sub-dimensions of interpersonal harmony in class were significantly negatively related to both internalizing and externalizing problems, highlighting the potential protective role of a supportive environment at school.

Partially consistent with our hypothesis II, moderation analysis revealed the moderating effects of both teacher interpersonal climate and peer interpersonal climate on the association between parental rejection and externalizing problems. Our findings suggest that the associations between perceived parental rejection and externalizing problems were weakened for those who recognized higher level of teacher interpersonal climate and peer interpersonal climate. At the same time, we found that the moderating effects were significant only among the left-behind children with both parents absence, but not those with single parents absence, indicating the crucial protective role of school especially for left-behind children who suffer from both parents being away (Sun et al., 2015). However, our finding should be interpreted with caution considering the relatively small number of children with only father or mother absence compared to the large number of children with both parents absence in our study. It is therefore necessary for future studies to validate the difference between both parents absence vs. single parent absence.

For left-behind children, inadequate home supervision is a prevailing problem (Hung et al., 2023). However, when the class environment enables harmonious teacher-student or peer relationships, it provides behavioral models for left-behind children to regulate themselves. Meanwhile, it can be understood from the perspective of multiple attachment as a potential underlying mechanism (Charalampous et al., 2015). For this special group of children who are separated from parents for a long time, they typically lack secure attachment to their parents and stable interpersonal relationships (Li and Liu, 2013; Tan et al., 2023). When they are provided with good teacher-student relationships and peer relationships, even though neither of these relationships can fully counterbalance the parental absence, the negative effects of poor parent–child interactions (such as parental rejection) on left-behind children can be mitigated, especially for externalizing problems.

Contrary to our hypothesis II, we did not find the moderating effect of class interpersonal harmony on the association between parental rejection and internalizing problems, which may be due the fact that internalizing problems such as anxiety, depression or other emotional disturbances are inner-directed and normally less visible (Achenbach et al., 2016). Children with observable externalizing problems typically cause troubles and conflicts in the class environment, and are thus more likely to draw attention from their teachers or peers (Roorda and Koomen, 2021). Our finding implies that although school supervision may be beneficial to regulate observable externalizing problems, it is insufficient to buffer the negative impact of parental rejection on internalizing problems of left-behind children. On the other hand, parental rejection may impact children’s internalizing problems via the role of insecure attachment. As stated before, previous research has shown that left-behind children typically has insecure attachments to their parents (Chai et al., 2019; Li and Liu, 2013; Tan et al., 2023), while insecure parent–child attachment normally leads to higher risks of internalizing symptoms such as anxiety and depression in children and adolescents (Brumariu and Kerns, 2010). Studies in the future may examine the potential mediation role of insecure attachment in the relation between parental rejection and left-behind children’s internalizing problems.

We did not find the moderating effect of class structure on the relationship between parental rejection and internalizing/externalizing problems of the left-behind children. However, it is understandable considering the fact that teacher interpersonal climate (Li Y. et al., 2015) and peer interpersonal climate (Boivin et al., 2005) are direct interpersonal interactions within the class, which involves emotional interactions and affective aspects, whereas class structure refers to the construction or organization of student roles within the class, such as goal guidance or class management (Chen and Li, 2009). Example items in the class structure sub-scale include: “We provide suggestions for class activities”; “Students actively participate in class affairs and activities.” Therefore, this dimension assesses the constructive organizational behaviors in the class and may be less influential on left-behind children’s mental health and behavioral problems than the other dimensions in terms of teacher interpersonal climate and peer interpersonal climate.

Subgroup comparison found significant gender differences. Male left-behind children perceived more parental rejection than female left-behind children, which is consistent with some previous research findings (Ki et al., 2018). This may seem contradict the tradition of preference for sons in rural China (Wang et al., 2020). However, it is instead understandable considering the context of left-behind children. Boys, who are assumably favored by their parents, may have stronger feelings of parental rejection when being left behind, while girls, who are relatively devalued by their parents, may feel less parental rejection. In addition, the peer interpersonal climate and class structure perceived by male left-behind children were significantly lower than those of female left-behind children. This can be attributed to the fact that girls are typically more involved in classroom management and are better at maintaining good relationships with their teachers and classmates (Chen et al., 2005). Finally, male left-behind children had significantly more externalizing problems than female left-behind children, which is consistent with the perception that boys are more mischievous than girls as well as evidence from previous studies (Chi and Cui, 2020; Hicks et al., 2007).

This study has some limitations. Firstly, its cross-sectional design does not allow us to draw causal conclusions. A longitudinal design is warranted to examine the progression of left-behind children’s internalizing/externalizing problems, as well as the causal relationships between parental rejection, interpersonal harmony in class, and internalizing/externalizing problems. Moreover, it is possible that parental rejection and class interpersonal harmony do not constitute independent effects. For example, parental rejection might lead to insecure attachment and thus negatively influence children’s ability to establish stable interpersonal relationships, therefore undermining children social competence to form class interpersonal harmony. Meanwhile, the relationship between interpersonal factors at school and behavioral adjustment (i.e., internalizing and externalizing problems) may not be unidirectional, but rather interactive (e.g., Yang et al., 2020). It is mandatory for further studies to consider and examine the mutual influential nature of the variables. Secondly, although the sample size of this study was relatively large, the study sample was all from one rural area in the southwest China, and therefore the generalization of our findings should be made with caution. A large-scale study covering all rural areas of China would be advantageous to reflect a more comprehensive picture of left-behind children in China. Thirdly, this study only focused on left-behind children. Although they comprise a typical sample to investigate the effect of parental rejection, it remains unclear how parental rejection and class interpersonal harmony affect normal children or any other socioeconomically disadvantaged children. It would be beneficial for future studies to compare left-behind children with other children to reveal the relationship between interpersonal dynamics and behavioral adjustment by using diverse samples.

Despite these limitations, this study provides valuable data to understand internalizing and externalizing problems among the left-behind children in China, bearing implications for interventions and policy making. Ideally, the entire society should strive to tackle the problem at the root, i.e., having children migrate to urban areas together with their parents, or developing the rural economy to provide more employment opportunities and higher income to reduce regional disparity so that parent could work and live in local areas. High quality parent–child relationships can lay the foundation for children’s healthy development and minimize the risks of internalizing/externalizing problems (Zhou et al., 2020). Therefore, at the micro- and meso-levels, it would be beneficial to offer psychological education and support to parents so that they become aware of the detrimental effect of parental rejection, which is perceived rather normal in traditional Chinese culture (Ma et al., 2024). It would also be advantageous for schools and teachers to encourage increased parental involvement in left-behind children’s development as well as to facilitate a harmonious class climate. At the same time, at the macro- and meso-levels, local governments and social organizations can formulate relevant policies and intervention programs to support rural schools in building interpersonal harmony in classes and to strengthen teacher-student and peer relationships for left-behind children, thereby reducing risks of internalizing and externalizing problems of left-behind children caused by parental rejection.

## 5 Conclusion

In summary, the results show that parental rejection perceived by left-behind children in rural China was positively associated with their internalizing and externalizing problems, and that interpersonal harmony in class, teacher interpersonal climate and peer interpersonal climate could mitigate the detrimental effect of the deficiency of parental warmth and acceptance on left-behind children’s externalizing problems. Our findings suggest that the construction of a good teacher interpersonal climate and peer interpersonal climate can reduce the externalizing problems of left-behind children. Therefore, when the optimal solutions are unrealizable in the short-term, strengthening left-behind children’s interpersonal connections with their teachers and peers could be a promising strategy for schools, local governments and social organizations to resolve to.

## Data Availability

The raw data supporting the conclusions of this article will be made available by the authors, without undue reservation.
